# Bootstrapping promotes the RSFC‐behavior associations: An application of individual cognitive traits prediction

**DOI:** 10.1002/hbm.24947

**Published:** 2020-03-16

**Authors:** Lijiang Wei, Bin Jing, Haiyun Li

**Affiliations:** ^1^ School of Biomedical Engineering Capital Medical University Beijing China; ^2^ Beijing Key Laboratory of Fundamental Research on Biomechanics in Clinical Application Capital Medical University Beijing China

**Keywords:** bootstrapping, cognitive trait, feature selection, functional connectivity, individualized prediction

## Abstract

Resting‐state functional connectivity (RSFC) records enormous functional interaction information between any pair of brain nodes, which enriches the individual‐phenotypic prediction. To reduce high‐dimensional features, correlation analysis is a common way for feature selection. However, resting state fMRI signal exhibits typically low signal‐to‐noise ratio and the correlation analysis is sensitive to outliers and data distribution, which may bring unstable features to prediction. To alleviate this problem, a bootstrapping‐based feature selection framework was proposed and applied to connectome‐based predictive modeling, support vector regression, least absolute shrinkage and selection operator, and Ridge regression to predict a series of cognitive traits based on Human Connectome Project data. To systematically investigate the influences of different parameter settings on the bootstrapping‐based framework, 216 parameter combinations were evaluated and the best performance among them was identified as the final prediction result for each cognitive trait. By using the bootstrapping methods, the best prediction performances outperformed the baseline method in all four prediction models. Furthermore, the proposed framework could effectively reduce the feature dimension by retaining the more stable features. The results demonstrate that the proposed framework is an easy‐to‐use and effective method to improve RSFC prediction of cognitive traits and is highly recommended in future RSFC‐prediction studies.

## 1 INTRODUCTION

Resting‐state functional connectivity (RSFC) has been found to be associated with pathological conditions and physiological status of the human brain (Cribben, Haraldsdottir, Atlas, Wager, & Lindquist, 2012; Elanbari et al., 2014; Finn et al., 2015; Meskaldji et al., 2016), which therefore could be used to understand individual differences in cognition and behavior (Jiang et al., 2018). Previous studies have reported that FC patterns could be used to predict individual behavioral and cognitive phenotypes, such as attention ability (Yoo et al., 2018), verbal creativity (Sun et al., 2019), intelligence ability (Finn et al., 2015; Jiang et al., 2019), and chronological age (Dosenbach et al., 2010; Liem et al., 2017). To model the RSFC‐phenotype association, four regression algorithms including connectome‐based predictive modeling (CPM), support vector regression (SVR), least absolute shrinkage and selection operator (LASSO), and Ridge regression have been frequently adopted for their good performances and interpretabilities (Coloigner, Phlypo, Bush, Lepore, & Wood, 2016; Cui & Gong, 2018; Dadi et al., 2019; de Vos et al., 2018; Gao, Greene, Constable, & Scheinost, 2019; Jiang et al., 2019; Meng et al., 2017; Ryali, Chen, Supekar, & Menon, 2012; Shen et al., 2017; Toiviainen, Alluri, Brattico, Wallentin, & Vuust, 2014). CPM is in fact a linear regression model and has been successfully applied in predicting intelligence (Beaty et al., 2018; Finn et al., 2015; Jiang et al., 2019), attention (Rosenberg, Finn, Scheinost, Constable, & Chun, 2017), cocaine abstinence (Yip, Scheinost, Potenza, & Carroll, 2019), and reading comprehension (Jangraw et al., 2018). SVR implements space transformation by some kernel function in order to achieve better prediction (Basak, Pal, & Patranabis, 2007), and has been utilized in prediction of mental disease (Rizk‐Jackson et al., 2011), brain maturity (Dosenbach et al., 2010; Nielsen et al., 2019), and painful sensation (Tu et al., 2015). LASSO provides sparse representation by driving redundant features to zero‐valued weights, and performs well in investigation of reward‐related behavior (Ferenczi et al., 2016), mental state (Haufe et al., 2014), temperament metrics (Jiang et al., 2018), and intelligence quotient (Jiang et al., 2019). Ridge regression adds an L2 penalization on the object function to shrink the regression coefficients, and shows good prediction performances in age (Khosla, Jamison, Kuceyeski, & Sabuncu, 2019), behavioral measures, and cognitive traits (Cui & Gong, 2018; Gao et al., 2019).

For the high‐dimensional RSFC, feature reduction is indispensable since redundant or irrelevant information may confound the statistical testing significance (Bunea et al., 2011), worsen the machine learning model performance (Arbabshirani, Plis, Sui, & Calhoun, 2017; Duangsoithong & Windeatt, 2010), and increase the computational complexity. For regression tasks, correlation analysis between RSFC and the target phenotypic measure (e.g., cognitive trait) is the commonest approach to select effective features (Dadi et al., 2019; Gabrieli, Ghosh, & Whitfield‐Gabrieli, 2015). However, the correlation analysis may not be able to catch the reliable correlational relationship since resting state fMRI (rs‐fMRI) data exhibit low signal‐to‐noise ratio (SNR) and the correlation analysis is sensitive to outliers (Wilcox, 2004, 2005). Small perturbation in the data may lead to inclusion of unrelated features or exclusion of useful features, thus the weak robustness of correlation analysis can introduce false correlations including Type I error and power problem (Rousselet & Pernet, 2012). In addition, data distribution may be another easily ignored factor in the correlation analysis: it is also susceptible to the clustered points, curvature, heteroscedasticity, and range (Rousselet & Pernet, 2012). All these conditions may cause that the features detected from the whole dataset do not significantly correlate with the target phenotypic measure in a subset, resulting in unstable features, which may in fact contribute little to the target phenotypic prediction. Thus, an adequate feature selection method that could detect intrinsic stable RSFC features is of great importance to the prediction task.

Bootstrapping is a statistical method that relies on the random sampling (Efron & Tibshirani, 1997; Hall & Robinson, 2009), and any focused statistical test can be examined in newly resampled datasets. After repeating the resampling process several times, the stability of the statistical significance in each resampled dataset could be accessed, which could be finally used to ascertain the statistical test by setting a specific threshold. The main advantage of bootstrapping is that it does not rely on any assumption on the data, so it can ascertain the actual and stable between‐group statistical significance. A few literatures have adopted bootstrapping to investigate the stability of selected features. In Alonso‐Atienza et al. (2012), the bootstrapping was used as a backward feature selection method for cardiac ventricular fibrillation discrimination. Ditzler reported that the bootstrapping combined with Neyman–Pearson hypothesis test successfully detected the statistically relevant features on both synthetic and real data (Ditzler, Polikar, & Rosen, 2015). In Bunea et al. (2011), features which were selected most frequently by penalized least‐squares regression methods in bootstrapping resamples were identified as useful predictors in neuroimaging. However, few studies tried to explore the influence of different parameter settings on the bootstrapping. For example, Bunea et al. (2011) used the bootstrapping with only one cutoff threshold (50% inclusion frequency) to derive the features. Abram et al. (2016) proposed a quantile threshold bootstrapping method for feature selection in penalized regression models. Hence, studies were highly needed to make known how different parameter settings in the bootstrapping influenced the final prediction performance.

This study explored a bootstrapping‐based feature selection framework to enhance the RSFC prediction performances in CPM, SVR, LASSO, and Ridge models, and different parameter settings in bootstrapping were compared in order to ascertain the optimal parameter setting, which mainly included two categories: bootstrapping without replacement (i.e., subsample without replacement) and bootstrapping with replacement. To verify these bootstrapping‐based feature selection methods, a large public dataset from Human Connectome Project was used in this study, and 13 cognitive traits were selected as the prediction targets. We hypothesized that bootstrapping could improve the RSFC prediction performances of CPM, SVR, LASSO, and Ridge models.

## 2 MATERIALS AND METHODS

### 2.1 Resting‐state fMRI data

The data came from public WU‐Human Connectome Project (HCP) 1,200 release of 1,206 healthy subjects (age 22–37). All imaging data were collected on a customized 3T ConnectomeScanner adapted from a Siemens Skyra (Siemens, Erlangen, Germany) scanner using a standard 32‐channel head coil. Rs‐fMRI data were acquired with a multiband pulse sequence with the following scanning parameters: time repetition (TR) = 720 ms, time echo = 33.1 ms, field of view = 208 × 180 mm^2^, flip angle = 52°, and voxel size = 2.0 mm isotropic cube (Smith et al., 2013). Two scanning sessions (REST1 and REST2) of high spatial–temporal resolution rs‐fMRI data were acquired during two consecutive days. Both sessions were scanned at right‐to‐left (run1) and left‐to‐right (run2) phase encoding direction, and the functional images comprised 1,200 volumes in each run. The current study adopted the 1,003 subjects with complete rs‐fMRI runs (a total of 4,800 time points). Note that the HCP database has two types of reconstruction data: 191 subjects were reconstructed utilizing an original reconstruction algorithm (recon1) that performed separation of the multiband multi‐slice images after transforming the acquired fully sampled data to frequency space along the readout direction, while the other 812 subjects applied an upgraded algorithm (recon2) that performed the separation in k‐space. To avoid possible influences caused by reconstruction methods and validate the generalization of the proposed bootstrapping methods, we used 812 (recon2) subjects as a discovery dataset, and the remaining 191 (recon1) subjects as an independent validation dataset. For more details of inclusion and exclusion criteria on the HCP dataset, please see Van Essen et al. (2013).

### 2.2 Preprocessing

The extensively preprocessed PTN (Parcellation+ Timeseries+ Netmats) data were utilized in our study. Details of preprocessing steps could be found in Smith et al. (2013). In brief, each run of rs‐fMRI data underwent a minimal preprocessed pipeline and ICA+FIX to remove the potential artifacts. All four runs were concatenated to form 4,800 volumes for each individual. After region parcellation by group ICA, the subject‐specific FC matrices, or connectomes, were calculated using Pearson's correlation and then Gaussianized into Z‐stats. Here, we adopted 300 ICA components as network nodes, resulting in a connectivity matrix with size 300 × 300 for each participant.

### 2.3 Cognitive traits

Given that the cognitive activities were functionally modulated by the brain, prediction of cognitive traits by RSFC was a feasible task (Sripada et al., 2019; Stevens, 2009). Previous studies had shown that RSFC patterns were closely associated with cognitive traits (Santarnecchi, Galli, Polizzotto, Rossi, & Rossi, 2014; Sun et al., 2019). In this study, 13 measures of cognitive tests were chosen as the prediction targets that provided by HCP (Kong et al., 2019; Li et al., 2019). These tests included Picture Sequence Memory, Dimensional Change Card Sort, Flanker Task, Oral Reading Recognition, Picture Vocabulary, Pattern Completion Processing Speed, List Sorting, Penn Progressive Matrices, Delay Discounting, Variable Short Penn Line Orientation, Short Penn Continuous Performance, and Penn Word Memory (Table S1). Seven of them measured by NIH (National Institutes of Health) Toolbox were normalized to age‐adjusted scores with mean of 100 and *SD* of 15. Furthermore, some subjects with the missing cognitive measures were excluded in corresponding trait prediction analysis (see Table S1 for missing subjects).

### 2.4 Bootstrapping‐based feature selection methods

**Figure 1:**
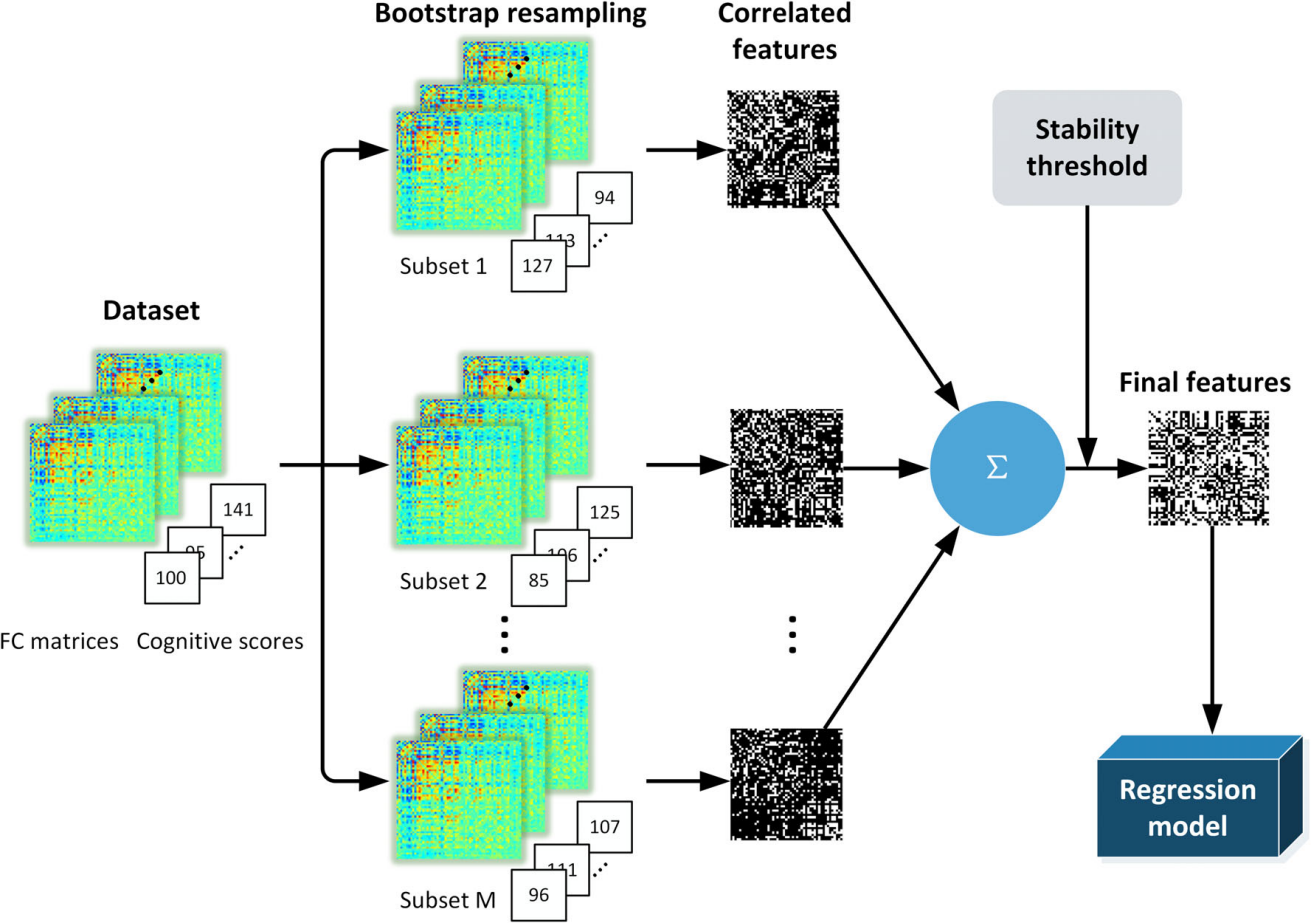
The pipeline of bootstrapping framework

The bootstrapping‐based feature selection methods sought to find stable features that were consistently identified in the resampling subsets. Conventional bootstrapping method mainly focused on resample with replacement, but it usually required a high amount of resample times, which seemed not efficient enough for feature selection. Moreover, medical imaging dataset was usually not large enough, in this condition, bootstrapping without replacement might be more suitable to the finite population. In this study, both bootstrapping with and without replacement (Figure 1) were investigated, and a wide range of parameter settings on bootstrapping (with and without replacement) were tested and evaluated to make sure the optimal parameter setting of bootstrapping.

#### 2.4.1 Bootstrapping without replacement

Bootstrapping without replacement took out samples without replacement from an original dataset, so each subject in the resampling dataset was unique without duplication. For each cognitive measure, the bootstrapping without replacement was applied on the dataset to extract the feature vector. Let NB (number of bootstrapping) denote the sampling times. Within each resampling iteration, a portion of subjects were randomly selected without repetition. The proportion of sampling was defined as bootstrap percentage (BP), for example, BP = 50% indicated that half of all subjects were chosen from the original dataset to form a resample subset. Spearman correlation analysis was then conducted in each resampling set between RSFC and cognitive measure, and significant features were selected as predictors. After all bootstrapping iterations finished, a stability threshold for frequency percentage (FP) of feature was used to determine final feature sets in NB bootstrapping datasets. For example, if the stability threshold was set to 50%, then features whose FP more than 50% would be selected into the final feature set, otherwise it would be filtered as weak predictors.

#### 2.4.2 Bootstrapping with replacement

In the bootstrapping with replacement method, resamples were obtained with replacement from the original samples, so the sample size of resampling subset was same as the original dataset size. There were only two parameters in this method, that is, NB and FP. Likewise, after performing Spearman correlation analysis in NB bootstrapping subsets, features with FP larger than stability threshold were chosen as predictors.

### 2.5 Regression models

Four widely used regression models were employed in the study to predict the cognitive traits with RSFC, including CPM, SVR, LASSO, and Ridge regression.

#### 2.5.1 Connectome‐based predictive modeling

Briefly, CPM summarized the relevant RSFCs for each individual and fitted the summary values with cognitive measures in a linear regression model. The detail steps of CPM were as follows:

1. Find features in RSFC matrices that correlated with the cognitive measure, and significantly positive or negative features were respectively selected.
2. Summarize selected feature values of positive feature set and negative feature set separately for each individual.
3. Perform univariate linear regression between the summary value and the cognitive measure for positive and negative feature sets separately.
4. Test the model performance with the unseen testing data.

Here, label the CPM model using positive or negative feature sets respectively as CPM‐P or CPM‐N.

#### 2.5.2 Support vector regression

SVR was based on statistical learning theory and fitted linear model with Vapink's ε‐sensitive loss function (Smola & Scholkopf, 2004). It tried to solve an objective function *f*(*x*) whose predicted values of training data deviated by no more than *ε* from the actual values and flatness of regression line was maximized. Samples that deviated by more than *ε* from their actual values were called support vectors. Given *N* training samples ((*x*_1_, *y*_1_), …, (*x*_*N*_, *y*_*N*_)), the object function took the following form:

$$\min_{\beta ,\xi_{i},{\hat{\xi }}_{i}}\frac{1}{2}{\left\|\beta \right\|}^{2}+C\sum_{i=1}^{m}\left(\xi_{i}+{\hat{\xi }}_{i}\right)$$

$$s.t.f\left(x_{i}\right)-y_{i}\leq \varepsilon +\xi_{i},$$

$$y_{i}-f\left(x_{i}\right)\leq \varepsilon +{\hat{\xi }}_{i},$$

$$\xi_{i},{\hat{\xi }}_{i}\geq 0$$

where *β* was the regression coefficient vector for features, *m* was the number of support vectors, *ξ*_*i*_ and ${\hat{\xi }}_{i}$ were slack variables, and *C* was the penalty parameter that controlled the trade‐off of penalty between variance and bias. The object function was solved to obtain a weight for each support vector. In this study, the linear kernel was selected for SVR.

#### 2.5.3 Least absolute shrinkage and selection operator

LASSO was a penalized regression method by adding L1‐norm regularization to ordinary least squares (Tibshirani, 1996). The method could attenuate regression coefficients of correlated features except one feature among them to zeros (Zou & Hastie, 2005), thus generating robust coefficients as well as parsimonious models. This algorithm minimized the ordinary least squares plus the sum of absolute values of regression coefficients *β* to obtain the estimated coefficients. The object function was formed as below:

$$\min_{\beta }\sum_{i=1}^{N}{\left(f\left(x_{i}\right)-y_{i}\right)}^{2}+\lambda \sum_{j=1}^{P}|\beta_{i}|$$

where *λ* was the tuning parameter which controlled balance between accuracy and sparsity.

#### 2.5.4 Ridge

Ridge applied L2‐norm regularization on ordinary least squares, which had the ability to shrink regression coefficients. The model minimized ordinary least squares and the L2‐norm regularization, and the object function could be formulized as below:

$$\min_{\beta }\sum_{i=1}^{N}{\left(f\left(x_{i}\right)-y_{i}\right)}^{2}+\lambda \sum_{j=1}^{P}{\left\|\beta_{i}\right\|}^{2}$$

where *λ* controlled the trade‐off between accuracy and shrinkage strength.

At last, CPM, SVR, LASSO, and Ridge regression accompanied with correlation analysis on the dataset were marked as the baseline method, and the corresponding models combined with the bootstrapping‐based feature selection were regarded as the proposed methods.

### 2.6 Prediction framework

For each cognitive trait, a 10‐fold cross‐validation (CV) framework was utilized in the discovery dataset. All individuals in the discovery dataset were sorted according to values of cognitive measure and then divided into 10 folds. To be specific, the (1st, 11th, 21st,…) subjects were assigned to the 1st fold, the (2nd, 12th, 22nd,…) subjects were assigned to the 2nd fold, and so on. This partition procedure ensured the same distribution of the 10 folds and avoided random bias and expensive computation due to random splitting (Cui & Gong, 2018). Within the prediction framework, each fold was iteratively used for testing while the remaining ninefolds were used for training. The bootstrapping feature selection methods were performed on the training set to select features for CPM, SVR, LASSO, and Ridge models (Figure 1). Significantly positive‐, negative‐, and combined‐correlated FCs were allotted into positive, negative, and combined feature set (significant threshold *p* = .05). Then, the positive feature set was adopted for CPM‐P, the negative feature set for CPM‐N, and the combined feature set for SVR, LASSO, and Ridge. In both bootstrapping method with and without replacement, a broad sets of bootstrapping parameters including NB, BP, and FP were tested, where NB was chosen from [10, 20, 50, 100, 500, 1,000] and FP from [50, 60, 70, 80, 90, 100%]. Besides, in bootstrapping without replacement, BP was chosen from [25, 50, 60, 70, 80%]. All parameter settings resulted in 180 combinations in bootstrapping without replacement and 36 combinations in bootstrapping with replacement. For SVR, LASSO and Ridge, inner fivefold CVs were employed to determine their optimal model parameters (i.e., *C* and *λ*). Parameter *C* of SVR ranged from [2^−5^, 2^−4^,…, 2^10^] and parameter *λ* of LASSO and Ridge ranged from [2^−10^, 2^−9^,…, 2^5^] (Cui & Gong, 2018). The model performance was evaluated by the correlation value (*R*) and the mean square error (*MSE*) between predicted and actual cognitive values. In our study, the reported correlation *R* and *MSE* were averaged over 10 testing folds. Among all candidate parameter combinations, the prediction result with the largest *R* was served as the optimal bootstrapping parameter setting.

### 2.7 Feature dimension reduction

To illustrate the feature dimension reduction with bootstrapping‐based feature selection methods, the number of features selected by baseline method and bootstrapping methods was respectively compared. For each cognitive trait, the feature dimension was obtained by counting the feature number on each fold and then averaged across 10 folds. The final feature dimension was averaged across 13 cognitive traits to gain the mean feature dimension. Particularly, for the bootstrapping methods, only the feature dimension derived from the optimal bootstrapping parameter settings would be considered.

### 2.8 Computational cost

Since the bootstrapping methods required multiple resampling on the original dataset, computational cost would be a noticeable concern especially for a large dataset. Among the bootstrapping parameters, concerning NB was the main source of computational cost, the running time of resampling and correlation test under each NB was quantified for bootstrapping methods. Because only the bootstrapping without replacement had BP, the running time in bootstrapping without replacement was recorded for each BP and then averaged under different NB. All procedures were carried out with a multi‐core CPU (Intel(R) Xeon(R) E5‐2630 v4 @2.20GHz, 10 cores, 20 threads) with 128 GB memory.

### 2.9 Evaluation in the validation dataset

In order to test the generalization of predictive models obtained from the discovery dataset, we applied the predictive models acquired from the discovery dataset to predict subjects in the validation dataset. Notably, the predictive model was obtained with the whole discovery dataset, and features were selected by bootstrapping without replacement and baseline method, respectively. The reason we only used bootstrapping without replacement was that it displayed better prediction performance and faster computation than bootstrapping with replacement in the discovery dataset. Moreover, to simplify the bootstrapping parameter settings in the prediction model, only eight parameter combinations were explored based on the obtained optimal parameter settings in previous section: NB [50, 100], BP [0.7, 0.8], and FP [0.8, 0.9]. Likewise, the prediction performance was assessed by the correlation *R* and *MSE* between predicted and actual cognitive values.

## 3 RESULTS

### 3.1 Bootstrapping improved prediction of cognitive traits

**Figure 2:**
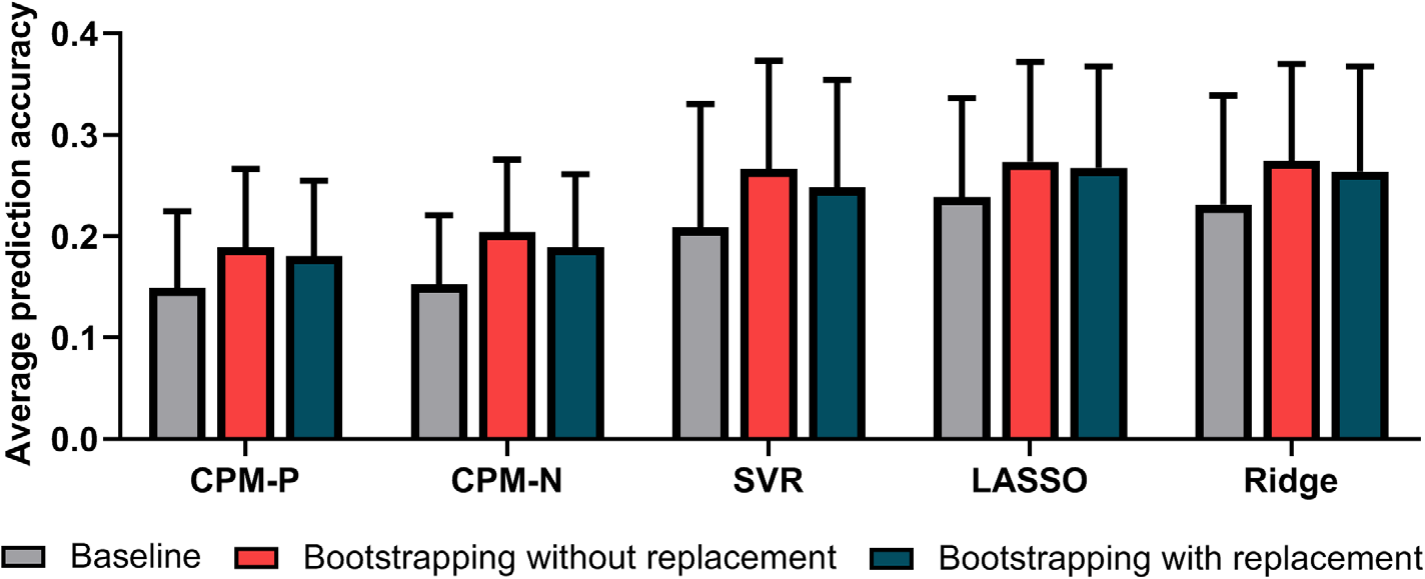
The mean and standard error of correlation *R* across 13 cognitive trait predictions. For bootstrapping methods, only the results derived from optimal parameter settings were plotted. Bootstrapping without replacement had the highest prediction accuracy. See Figure S1 for the corresponding mean square error (*MSE*)

**Figure 3:**
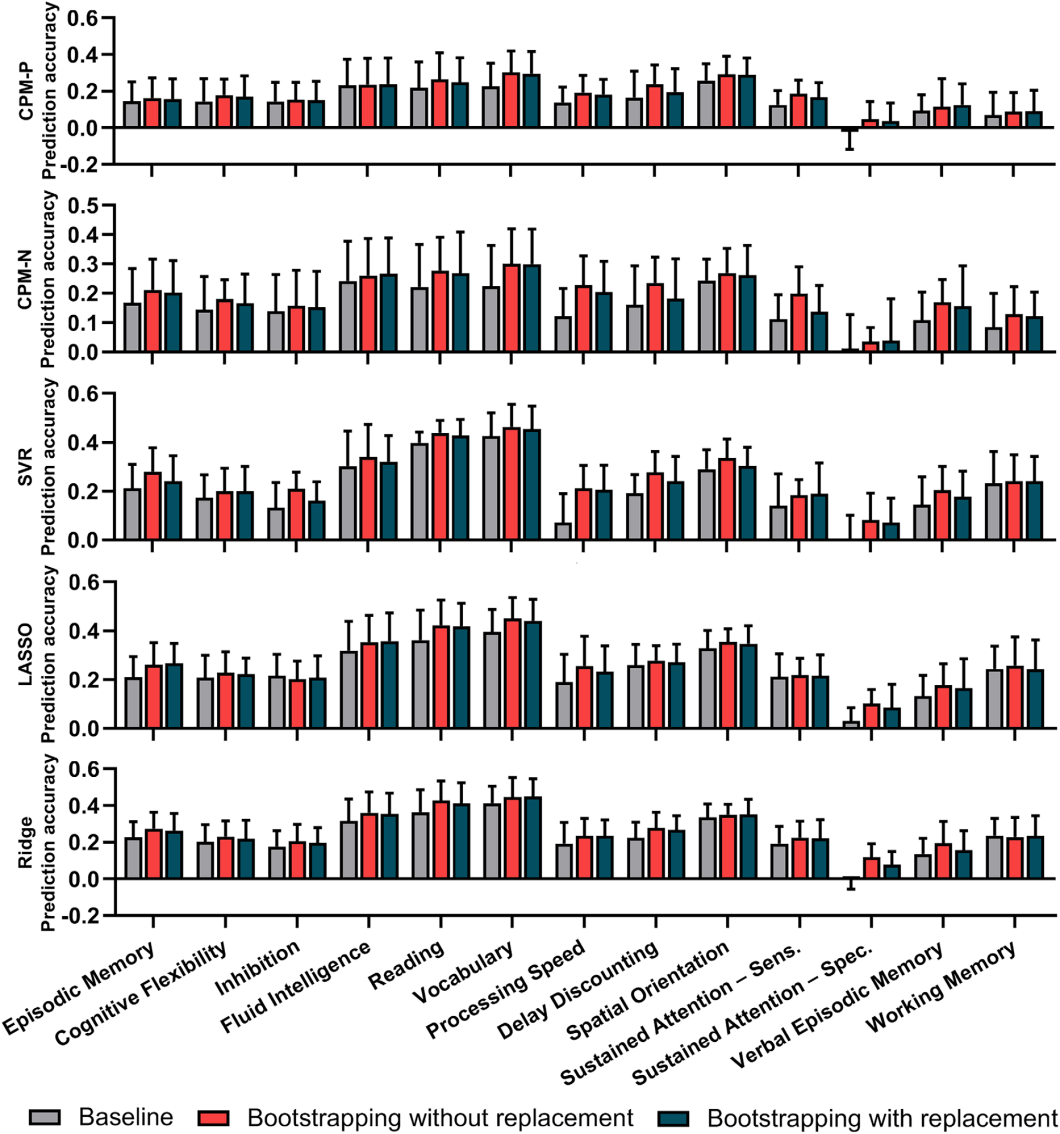
The correlation *R* of 13 cognitive trait predictions. The “bars” showed the mean and standard error of prediction correlation *R* across 10‐fold cross‐validation

Figure 2 summarized the mean correlation *R* (across 13 cognitive traits) of each model using the baseline method and bootstrapping method in discovery dataset, and only results derived from the optimal parameters were plotted for bootstrapping methods. All models using the refined feature sets derived from the optimal parameters outperformed the baseline method. Compared with the baseline method (mean correlation *R* = 0.15, 0.15, 0.21, 0.24, and 0.23 for CPM‐P, CPM‐N, SVR, LASSO, and Ridge, respectively), the bootstrapping without replacement method increased the predictive correlation values by 27.0, 33.6, 27.5, 14.8, and 18.5%. Among five models, SVR, LASSO, and Ridge performed best and the mean correlation *R* was up to 0.27, while CPM‐P and CPM‐N achieved similar performances of mean correlation *R* = 0.19 and 0.20, respectively. In the bootstrapping with replacement, the accuracy increased by 21.2, 23.9, 18.9, 12.1, and 14.0% for CPM‐P, CPM‐N, SVR, LASSO, and Ridge, respectively. Similar to the bootstrapping without replacement, the bootstrapping with replacement showed best accuracies in SVR (mean correlation *R* = 0.25), LASSO (mean correlation *R* = 0.27), and Ridge (mean correlation *R* = 0.26), and similar results in CPM‐P (mean correlation *R* = 0.18) and CPM‐N (mean correlation *R* = 0.19). The best prediction accuracies of 13 cognitive measures were illustrated in Figure 3 for bootstrapping with and without replacement. Figure S1 showed the *MSE* (across 13 cognitive traits) of each model using the baseline method and bootstrapping methods, and only results derived from the optimal parameters were plotted for bootstrapping methods.

### 3.2 Bootstrapping methods largely reduced feature dimension

**Figure 4:**
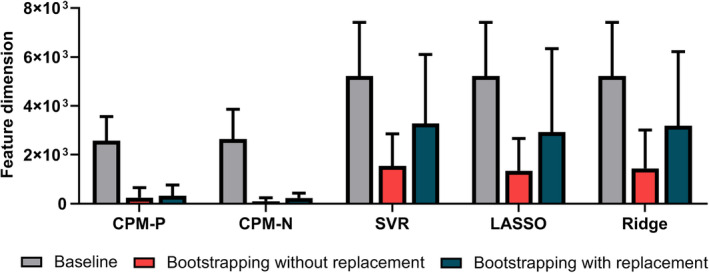
The comparison of mean feature dimension across 13 cognitive traits, where the feature dimension of bootstrapping methods were obtained under optimal parameter settings

**Figure 5:**
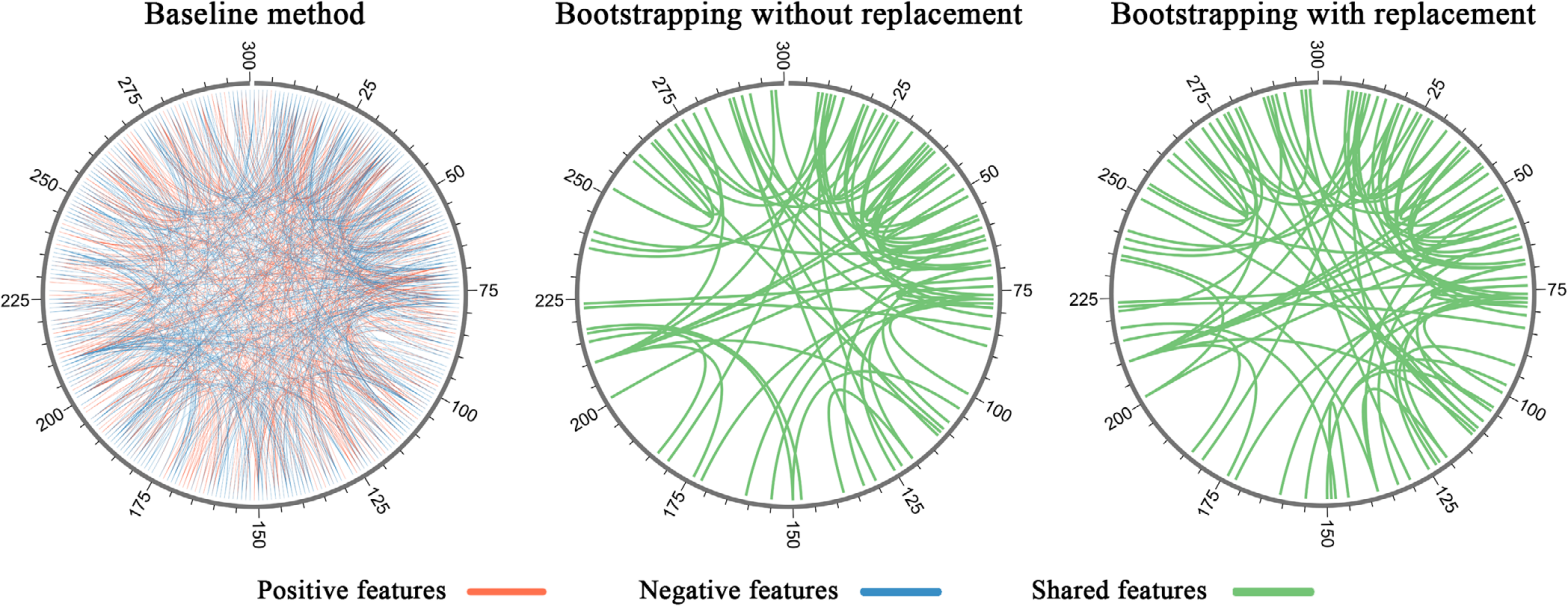
Resting‐state functional connectivity (RSFC) features of the Working Memory selected by the baseline method and the bootstrapping methods in all 10‐fold cross‐validation. Features selected by the bootstrapping methods were derived from NB = 100, BP = 70%, and FP = 80%. Orange and blue links denoted positive and negative features, respectively, and green links in bootstrapping methods denoted shared features, that is, features that were overlapping between the baseline method and each of bootstrapping methods. Note that all features selected by the bootstrapping methods overlapped with the baseline method

The feature dimension refined by the bootstrapping methods decreased enormously compared with the baseline method (Figure 4). Additionally, Figure 5 illustrated an example of features for Working Memory selected by the baseline method and bootstrapping methods. More importantly, the refined feature set by bootstrapping methods overlapped with feature set selected by baseline method (represented in green links). Using the bootstrapping without replacement, the average feature dimension decreased from 2,574 to 249 in CPM‐P, from 2,648 to 97 in CPM‐N, from 5,221 to 1,539 in SVR, from 5,221 to 1,342 in LASSO, and from 5,221 to 1,441 in Ridge. Using the bootstrapping with replacement, the average feature dimension decreased to 320 in CPM‐P, 235 in CPM‐N, 3,273 in SVR, 2,921 in LASSO, and 3,185 in Ridge. Generally, the bootstrapping without replacement reduced more features than bootstrapping with replacement.

### 3.3 Time consumption increased with the number of bootstrapping

**Figure 6:**
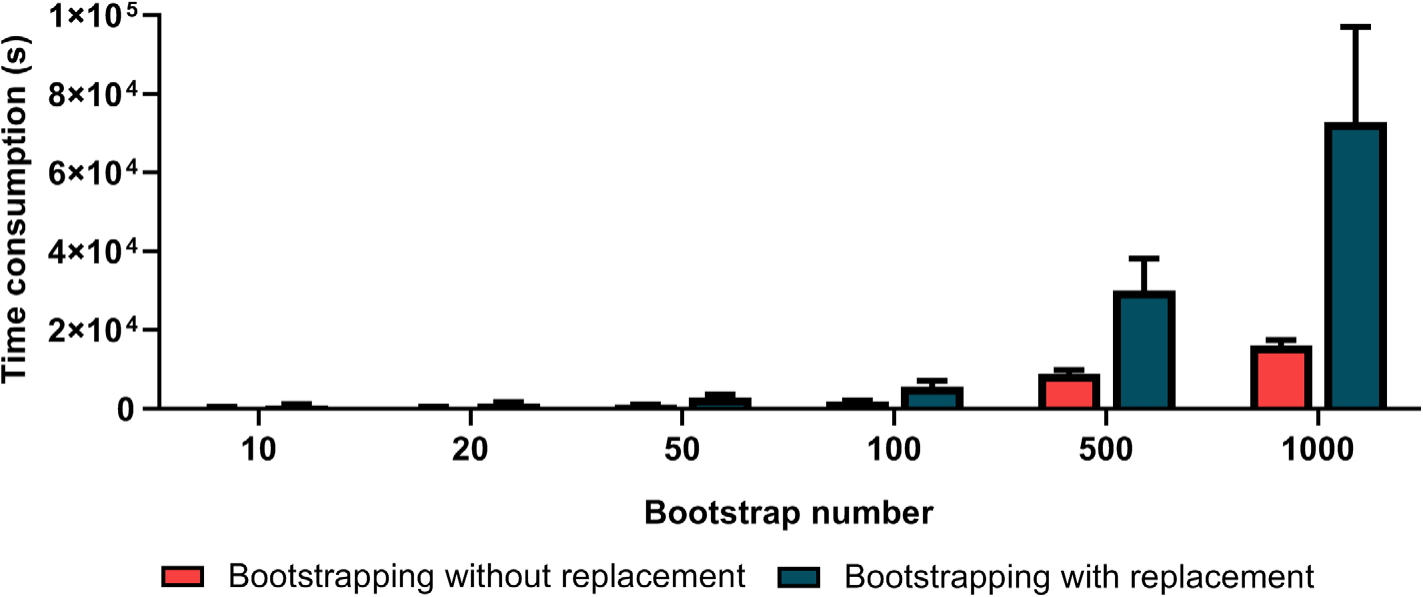
The mean time consumption of the bootstrapping methods across 13 cognitive traits

The time consumption of the bootstrapping methods gradually increased with NB (Figure 6). Under the same NB, bootstrapping with replacement consumed more time than bootstrapping without replacement. When NB exceeded 500, the average consumption time became significantly large, implying that NB was preferably set to be less than 500.

### 3.4 Optimal parameter settings were not constant for different cognitive traits

**Figure 7:**
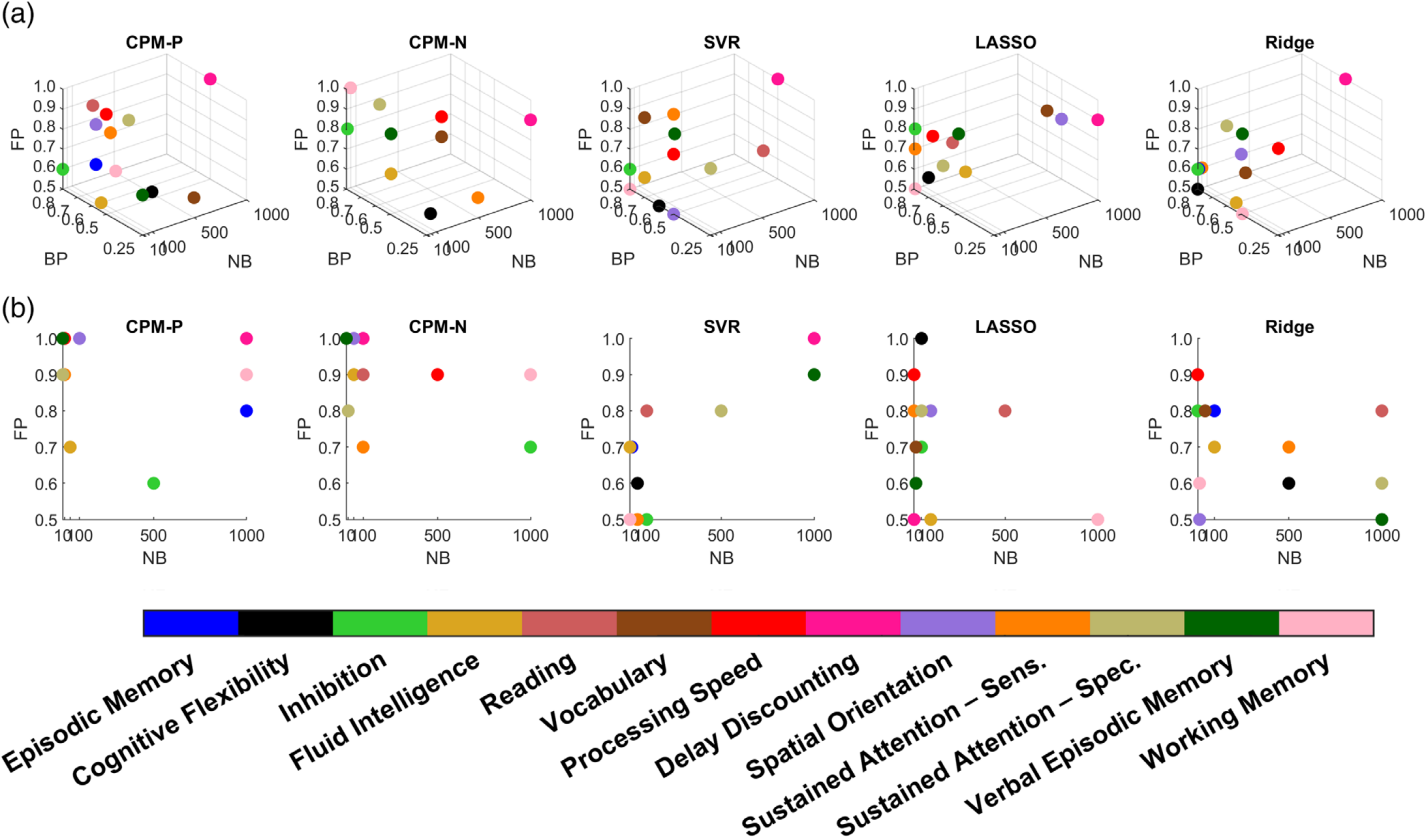
The optimal parameter setting for each cognitive measure in CPM‐P, CPM‐N, SVR, LASSO, and Ridge when using (a) bootstrapping without replacement and (b) bootstrapping with replacement. The colored dots denoted different types of cognitive scores

The optimal bootstrapping parameter settings (i.e., NB, FP and BP) for each cognitive measure were depicted in Figure 7. Here, a dot in the parameter space referred to the best parameter setting for one cognitive measure. The results showed that most of the values of the optimal NBs were small, and 72% of these were less than 100 in the bootstrapping methods. As for FP and BP, they varied in different models under different bootstrapping methods. In bootstrapping without replacement, most optimal FPs were relatively high (0.7~0.9) for CPM and LASSO, relatively small (around 0.5) for SVR, and moderate (0.5~0.7) for Ridge. In bootstrapping with replacement, most optimal FPs were relatively high (0.8~1.0) for CPM, LASSO, and Ridge, and moderate (around 0.5) for SVR, while relatively low BPs (around 0.5) were suitable for CPM, SVR, and Ridge, but moderate (around 0.8) for LASSO.

### 3.5 Bootstrapping also improved prediction of cognitive traits in the validation dataset

**Figure 8:**
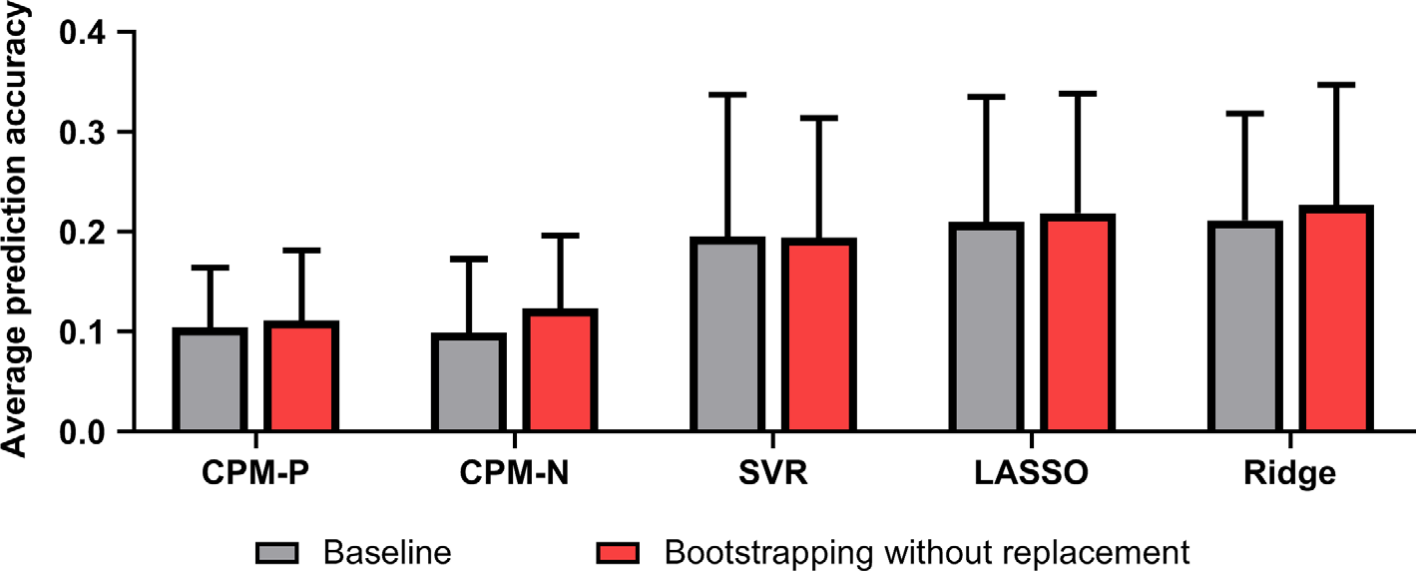
The mean correlation *R* averaged across 13 cognitive traits in the validation dataset using bootstrapping without replacement. The prediction accuracy was calculated by averaging the correlation *R* of each trait‐specific model across 8 predefined parameter settings and then averaging across 13 predictive models. See Figure S2 for the corresponding mean square error (*MSE*)

**Figure 9:**
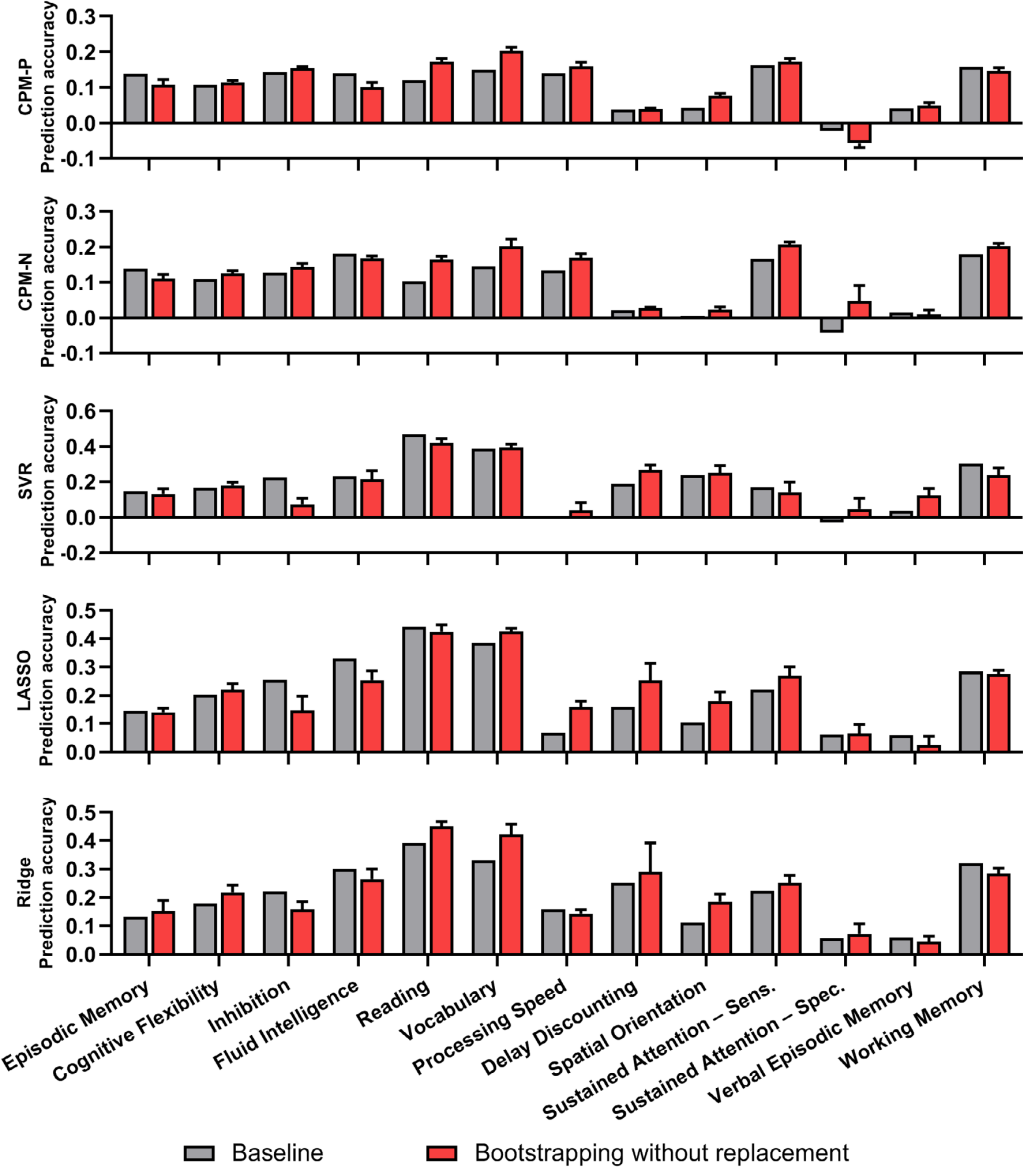
The correlation *R* of 13 cognitive trait predictions using the validation dataset. For bootstrapping without replacement, the “bars” showed the mean and standard error of correlation *R* across eight predefined parameter settings

Figure 8 illustrated the mean correlation *R* (across 13 cognitive traits) of each model using the baseline method and bootstrapping (without replacement) in the validation dataset. Compared with the baseline method (mean correlation *R* = 0.10, 0.10, 0.20, 0.21, and 0.21 for CPM‐P, CPM‐N, SVR, LASSO, and Ridge, respectively), the bootstrapping without replacement obviously increased the predictive correlation values by 5.8, 24.5, 4.3, and 7.2% for CPM‐P, CPM‐N, LASSO, and Ridge separately, while the mean correlation *R* of SVR remained almost unchanged. Among all models, LASSO and Ridge performed best and reached mean correlation *R* = 0.22 and 0.23, SVR achieved suboptimal performance at mean correlation *R* = 0.19, CPM‐P and CPM‐N achieved similar performances of mean correlation *R* = 0.11 and 0.12, respectively. The correlation *R* of 13 cognitive measures was illustrated in Figure 9, and mean *MSE* across 13 cognitive traits was shown in Figure S2.

## 4 DISCUSSION

In order to improve the performance of RSFC prediction, this study developed a bootstrapping based feature selection framework for RSFC prediction of cognitive traits. Using a large sample from HCP dataset, the prediction of cognitive traits with the bootstrapping methods significantly outperformed the baseline CPM, SVR, LASSO, and Ridge model under optimal parameters, and the dimension of selected features decreased dramatically using the bootstrapping methods. In addition, bootstrapping could improve the predictive performances in the validation dataset, demonstrating its good generalization.

### 4.1 Bootstrapping enhanced the RSFC‐phenotype associations

Modeling cognitive traits with RSFC is a persistent pursuit in modern cognitive neuroscience studies (Petersen & Sporns, 2015; Poldrack & Farah, 2015). A previous study showed that the diversity of individual cognition can be represented by the inter‐subject variations in the FC patterns (Mueller et al., 2013). However, the FC patterns may be noisy and unreliable (Braga & Buckner, 2017; Gordon et al., 2017; Gratton et al., 2018; Mueller et al., 2013), thus confounding the prediction precision. In recent years, improving RSFC‐phenotype association is a major concern in the domain, and several methodologies have been proposed from different perspectives. The first way is to use individual‐specific RSFC patterns to predict the behavioral traits. In Qin et al. (2019) and Kashyap et al. (2019), they decomposed RSFC (or timecourse) to extract individual‐specific RSFC (or timecourse) with different methods, which obtained obvious improvements in prediction. The second way is to combine various kinds of information with RSFC to enhance the prediction, such as task‐fMRI based FC (Elliott et al., 2019; Gao et al., 2019; Xiao, Stephen, Wilson, Calhoun, & Wang, 2019) and dynamic FC (Liegeois et al., 2019; Lim et al., 2018; Park et al., 2018), which could provide complementary information to the conventional FC. The third way is to decrease the influence of the possible noise in rs‐fMRI signal, for instance, the global signal regression (Li et al., 2019) and motion artifact correction (Nielsen et al., 2019) have been reported to advance the RSFC‐behavior prediction. The last way is to use the bagging strategy (Breiman, 1996) to improve the prediction with RSFC (Jollans et al., 2019).

Our proposed method provides another perspective to promote the RSFC‐phenotype prediction, and displays several advantages. First, the method does not need any collection of additional data (e.g., task fMRI) or any other processing (e.g., computation of dynamic FC and FC decomposition), so it is an easy‐to‐use and effective method. Second, the current method can purify the selected feature sets, making the prediction model more succinct and interpretable. It is known that reducing feature dimension is always beneficial to attenuate overfitting in neuroimaging data (Jollans et al., 2019). At last, previous studies (Alonso‐Atienza et al., 2012; Ditzler et al., 2015) used bootstrapping or bagging in a computation expensive manner, which requires the model construction and optimization in each bootstrapping resample dataset. This may lead to an unacceptable running time for the heuristic searching of optimal parameter setting in large neuroimaging dataset, and these studies had to use a constant parameter setting to conduct such methods without any optimal parameter searching. In contrast, our method is a more feasible manner and also displays good generalization that verified in the validation dataset.

As we know, good stability of biomarkers is thought to be as important as high classification performances (He & Yu, 2010). Currently, the importance of the stability of the rs‐fMRI signal (e.g., test–retest reliability) and the stability of selected biomarkers across sites (e.g., multicenter dataset) have been widely accepted (Marchitelli et al., 2016; Mueller et al., 2015; Noble, Scheinost, et al., 2017; Noble, Spann, et al., 2017), however, there is seldom study specially focusing on the stability of selected biomarkers across subsamples in one dataset. Indeed, heterogeneity exists in nearly all human neuroimaging datasets regardless of whether the participants are healthy or not, which may therefore result in instability in feature sets derived from subsamples and further weakens the prediction performance. It is necessary to eliminate the heterogeneity in the dataset and to obtain stable representations standing for neural activities. Figures 4 and 5 clearly showed that the amounts of fake correlated feature sets were large, implying that it was very beneficial to take into account the stability of feature selection for machine learning studies on neuroimaging dataset. Our study demonstrated that the proposed bootstrapping‐based framework is an effective way to eliminate the heterogeneity in the dataset and to obtain feature sets with good stability.

### 4.2 Bootstrapping with replacement versus bootstrapping without replacement

The bootstrapping with and without replacement were compared in the study. Although bootstrapping with replacement was referred more frequently than bootstrapping without replacement, it was reported not to guarantee reliable results sometimes. In Strobl, Boulesteix, Zeileis, & Hothorn (2007), for example, feature selection in random forest was biased by bootstrapping with replacement, while important measures of predictors could be accessed more reliably by bootstrapping without replacement. This phenomenon was not hard to explain, bootstrapping with replacement ensured the resample size as same as the original dataset, but the replication samples might introduce possible bias into the final feature selection, which was especially obvious in a finite dataset (e.g., neuroimaging data). For bootstrapping without replacement, the resamples was a subset of original dataset, so they could be regarded as sampling from the same population, and properties of statistical test in resamples were consistent with the original data (Rospleszcz, Janitza, & Boulesteix, 2016). In our study, bootstrapping without replacement selected less features and showed better prediction performances for cognitive measures than bootstrapping with replacement. Moreover, bootstrapping without replacement required less computational cost, because it resampled less samples from original dataset than bootstrapping with replacement did. Since bootstrapping without replacement demonstrated superiority in improving prediction performance, reducing redundant features and running cost than bootstrapping with replacement, we recommended bootstrapping without replacement as a feature selection technique to draw reliable predictors.

### 4.3 Comparison of CPM, LASSO, SVR, and Ridge regression

CPM had poorer prediction performance compared with SVR, LASSO, and Ridge models. There were three possible aspects accounted for it: (a) CPM is a simple linear regression model, while SVR, LASSO, and Ridge are models with L1 or L2‐norm regularization, which may therefore increase the model complexity and obtain better prediction performances. (b) The correlated feature sets were respectively divided into positive and negative feature sets in CPM, while SVR, LASSO, and Ridge used their combination, which may also weaken the CPM performance significantly. (c) CPM assigned same weight to all features, which might underestimate key predictors and overestimate weak predictors. However, when using bootstrapping methods, CPM achieved relatively large improvement in prediction and the most obvious reduction of features, suggesting future studies using CPM should consider the bootstrapping methods.

SVR could produce sparse model since it only relied on support vectors, and it could effectively decrease overfitting risk and display good generalization. However, efficiency was a crucial problem for high‐dimensional data while SVR was tremendously expensive in computation for model fitting (Cui & Gong, 2018; Shen et al., 2017).

LASSO ensured sparseness by randomly selecting one feature from correlated features, which was shown to perform well in challenging situation like potential features size exceeded the sample size (Abraham et al., 2017; Bunea, Tsybakov, & Wegkamp, 2007a, 2007b; Zhang & Huang, 2008). However, when confronting the condition that the feature number was remarkably larger than the sample size, it might meet such a problem: LASSO could only retain no more than *N* features (*N* is the sample size), thus some informative features might be discarded in the model (Efron, Hastie, Johnstone, & Tibshirani, 2004; Ryali et al., 2012).

Ridge regression can handle the problem of multicollinearity by adding a small positive quantity in the diagonal elements of design matrix (Hoerl & Kennard, 2000), whereas Ridge regression was unable to produce parsimonious model since it kept all predictors in the model without any selection (Zou & Hastie, 2005).

Although the four regression algorithms adopted were different, when combined with bootstrapping methods, the predictive models obtained higher prediction performances (Figure 2) and lower feature dimensions (Figure 3), suggesting that the benefits of the bootstrapping methods were not limited to specific regression algorithm. Besides, the bootstrapping methods displayed good generalization in the validation dataset, demonstrating bootstrapping methods should be adopted in future prediction tasks.

### 4.4 Optimal selection of parameter setting

There were three parameters for the bootstrapping methods (NB, BP, and FP), and it is not known beforehand which parameter setting was best for a give problem. In both bootstrapping methods, most of the best performances were achieved with NB less than 100 in discovery dataset, implying that the bootstrapping did not require a mass of resampling times to acquire stable features. Besides, small NB could lessen computational cost, so we recommended small NB (<100) for the bootstrapping methods. FP indicated the stability of the features in the resampling subset, thus specifying an appropriate threshold for FP was a key setting. In Bunea et al. (2011), the FP threshold was set to a single value (50%) in a bootstrapping‐based feature selection method in order to not miss any possibly relevant features, but our study found the optimal FP threshold varied for different cognitive traits, indicating that constant threshold setting could not guarantee the best performance. BP stood for the resample percentage of the bootstrapping without replacement from the original dataset, which also did not show obvious rules. The results from the validation dataset also demonstrated that although the optimal parameter setting for bootstrapping without replacement was not searched, it still enhanced overall prediction performances in different regression models. Therefore, in practical application, to identify optimal parameters is time consuming and seems unnecessary, to choose the parameters from a reasonable range would be also useful to achieve higher prediction accuracy.

### 4.5 Other methodological considerations

**Figure 10:**
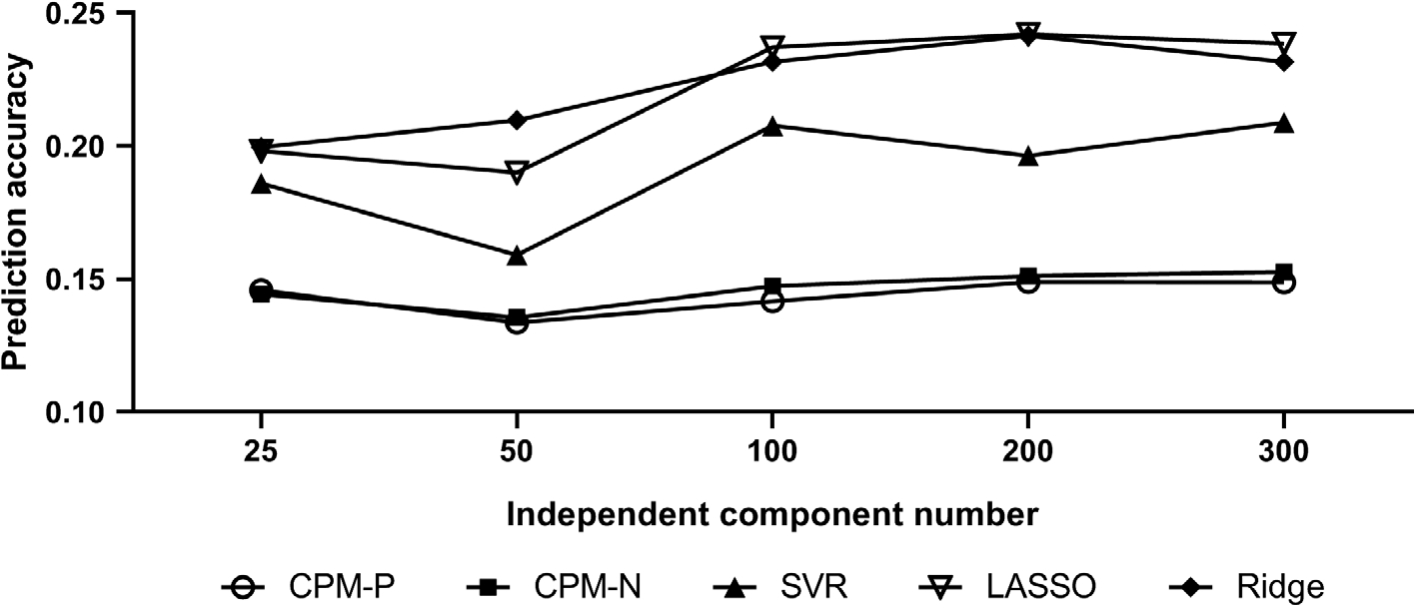
The prediction correlation *R* in different independent component number. Each point was mean correlation *R* averaging across 13 cognitive trait predictions, and only baseline method was used for feature selection. The result of 15 ICA components was not shown since no significant features were selected for some cognitive traits

Group ICA has been found to be a better way to define the functional brain nodes than the atlas‐based methods for its better prediction accuracy (Dadi et al., 2019), hence, group ICA parcellation was adopted to calculate the corresponding FC in the study. There were various brain parcellations derived from group ICA provided in HCP dataset, that is, [15, 25, 50, 100, 200, and 300]. To have direct comparisons among different parcellations, the baseline CPM, SVR, LASSO, and Ridge method were used to predict the cognitive traits with FC matrices under different parcellations. The results were illustrated in Figure 10, and the main tendency was that the prediction accuracy increased with component dimension, and grew slowly from 100 to 300 ICA components, and similar trend was also revealed in other studies (Abraham et al., 2017; Dadi et al., 2019). Therefore, the current analysis was only based on 300 independent components. Besides, significant threshold *p*‐value was an important parameter for correlation analysis. In order to conserve features as much as possible, the *p*‐value was set at .05 rather than other more stringent values in this study. In addition, concerning different head motion correction strategies and global signal regression were still controversial in functional connectivity studies, and we just focused on the bootstrapping related improvement, so we did not compare any other preprocessing steps on the preprocessed HCP data.

### 4.6 Limitation and future work

The bootstrapping methods were more expensive in computation compared with the baseline method, which could be speeded up via parallel computing with multiple CPUs, making the bootstrapping methods feasible in practical use. Although the bootstrapping methods could improve the RSFC‐phenotype association, the prediction accuracy was still not high enough, and future work should combine other improvement strategies together to make the RSFC‐based prediction applicable for clinical requirement. At last, although our study only focused on RSFC, the bootstrapping methods could easily extend to any correlation‐based prediction tasks with any imaging data, such as task‐fMRI functional connectivity, DTI and EEG.

## 5 CONCLUSION

This study presented a bootstrapping‐based feature selection framework and applied to CPM, SVR, LASSO, and Ridge models, and the results demonstrated the bootstrapping methods could not only improve the RSFC‐phenotype association but also purify the selected features into a lower dimension. Future RSFC prediction works were highly recommended to use the bootstrapping methods especially bootstrapping without replacement.

## AUTHOR CONTRIBUTIONS

L.W. performed the data analysis and wrote the draft. B.J. conceived and designed the experiments, and rewrote some paragraphs in Introduction and Discussion parts. H.L. revised the draft. All authors read, revised, and approved the final version of the manuscript.

## CONFLICT OF INTERESTS

The authors have no conflicts of interest to declare.

## Supporting information


**Table S1** The original HCP variable names with the corresponding descriptive labels used in the manuscript.
**Figure S1** The mean and standard error of MSE (mean square error) across 13 cognitive trait predictions. For the bootstrapping methods, only the results derived from optimal parameter settings were plotted. Bootstrapping without replacement had the highest prediction accuracy.
**Figure S2** The mean MSE (mean square error) averaged across 13 cognitive traits in the validation dataset. For bootstrapping without replacement, the prediction accuracy was calculated by averaging the *MSE* of each trait‐specific model across 8 predefined parameter settings and then averaging across 13 predictive models.

## Data Availability

Data were provided by the Human Connectome Project and WU‐Minn Consortium (Principal Investigators: David Van Essen and Kamil Ugurbil; 1U54MH091657) funded by 16 NIH Institutes and Centers that support the NIH Blueprint for Neuroscience Research; and by the McDonnell Center for Systems Neuroscience at Washington University. Code for this work is freely available at the GitHub repository (https://github.com/Lijiang-Wei/bootstrapping.git).
