# Supplementary material for: Bootstrapping promotes the RSFC‐behavior associations: An application of individual cognitive traits prediction
Source: Hum Brain Mapp. 2020 Mar 16;41(9):2302–16. doi: 10.1002/hbm.24947 (PMC7268063; doi:10.1002/hbm.24947)
Supplement: Supplementary file 1 — Table S1 The original HCP variable names with the corresponding descriptive labels used in the manuscript. Figure S1 The mean and standard error of MSE (mean square error) across 13 cognitive trait predictions. For the bootstrapping methods, only the results derived from optimal parameter settings were plotted. Bootstrapping without replacement had the highest prediction accuracy. Figure S2 The mean MSE (mean square error) averaged across 13 cognitive traits in the validation dataset. For bootstrapping without replacement, the prediction accuracy was calculated by averaging the MSE of each trait‐specific model across 8 predefined parameter settings and then averaging across 13 predictive models. [file HBM-41-2302-s001.docx]

**Table S1.** The original HCP variable names with the corresponding descriptive labels used in the manuscript.

| **Description** | **Test** | **HCP field** | **Missing subjects** |
| --- | --- | --- | --- |
| Visual Episodic Memory | Picture Sequence Memory | PicSeq_AgeAdj | 0 |
| Cognitive Flexibility | Dimensional Change Card Sort | CardSort_AgeAdj | 2 |
| Inhibition | Flanker Task | Flanker_AgeAdj | 0 |
| Fluid Intelligence | Penn Progressive Matrices | PMAT24_A_CR | 4 |
| Reading | Oral Reading Recognition | ReadEng_AgeAdj | 0 |
| Vocabulary | Picture Vocabulary | PicVocab_AgeAdj | 0 |
| Processing Speed | Pattern Completion Processing Speed | ProcSpeed_AgeAdj | 0 |
| Delay Discounting | Delay Discounting | DDic_AUC_40K | 3 |
| Spatial Orientation | Variable Short Penn Line Orientation Test | VSPLOT_TC | 3 |
| Sustained Attention – Sens. | Short Penn Continuous Performance Test | SCPT_SEN | 3 |
| Sustained Attention – Spec. | Short Penn Continuous Performance Test | SCPT_SPEC | 3 |
| Verbal Episodic Memory | Penn Word Memory Test | IWRD_TOT | 3 |
| Working Memory | List Sorting | ListSort_AgeAdj | 0 |


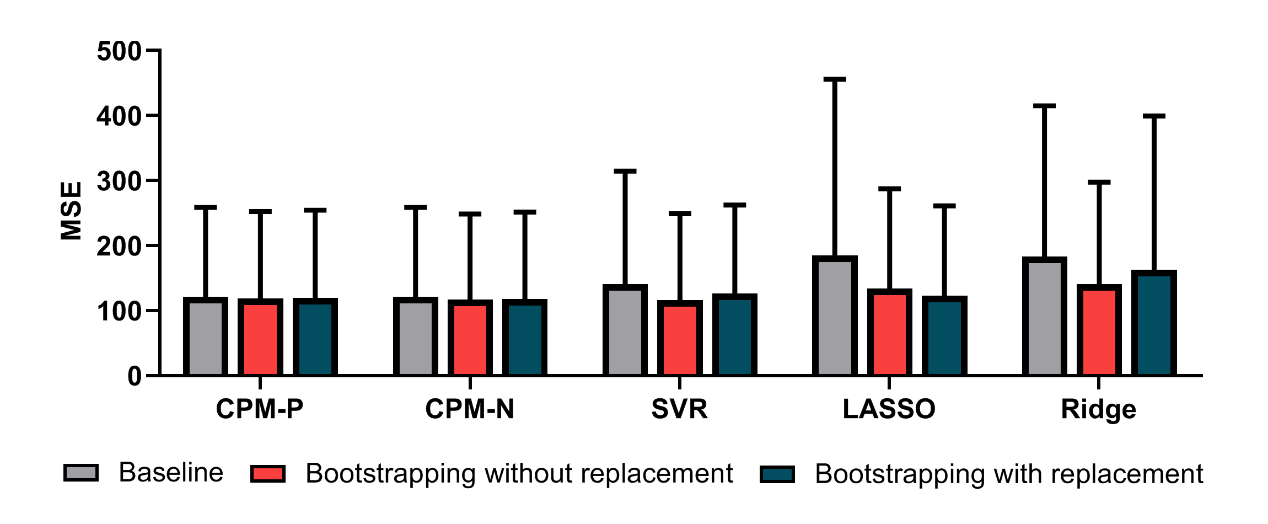


Figure S1. The mean and standard error of MSE (mean square error) across 13 cognitive trait predictions. For the bootstrapping methods, only the results derived from optimal parameter settings were plotted. Bootstrapping without replacement had the highest prediction accuracy.


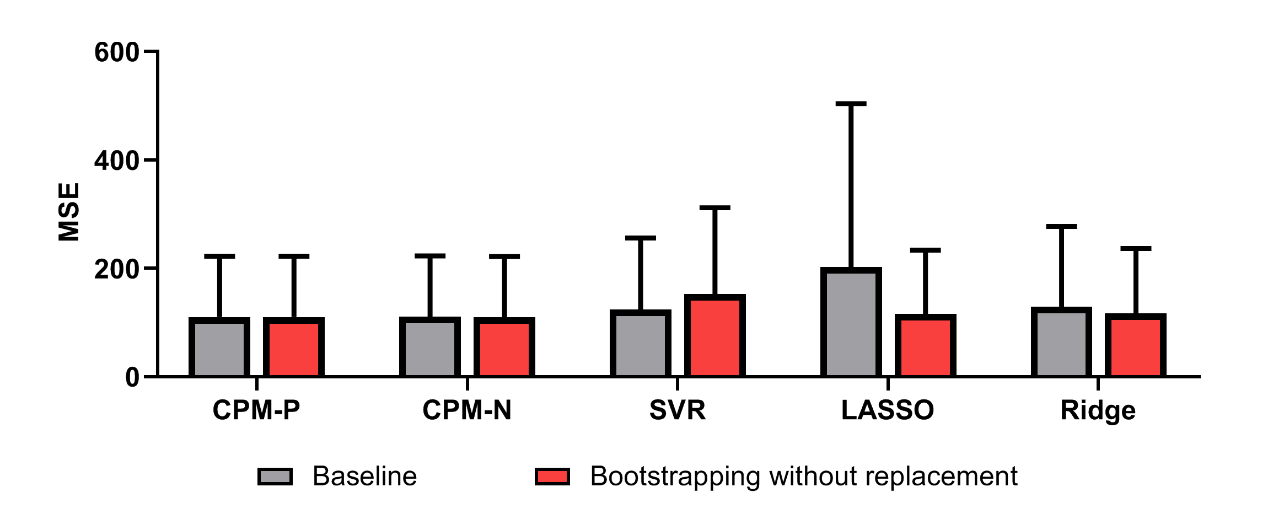


Figure S2. The mean MSE (mean square error) averaged across 13 cognitive traits in the validation dataset. For bootstrapping without replacement, the prediction accuracy was calculated by averaging the *MSE* of each trait-specific model across 8 predefined parameter settings and then averaging across 13 predictive models.
